# Lactate and Lactate Clearance Are Predictive Factors for Mortality in Patients with Extracorporeal Membrane Oxygenation

**DOI:** 10.21470/1678-9741-2023-0091

**Published:** 2024-02-06

**Authors:** Tamer Aksoy, Ahmet Hulisi Arslan, Murat Ugur, Hasim Ustunsoy

**Affiliations:** 1 Department of Anesthesiology and Reanimation, Anadolu Medical Center, Kocaeli, Turkey; 2 Department of Cardiovascular Surgery, Anadolu Medical Center, Kocaeli, Turkey; 3 Department of Cardiovascular Surgery, University of Health Sciences, Sancaktepe Sehit Professor Doctor Ilhan Varank Training and Research Hospital, Istanbul, Turkey

**Keywords:** Extracorporeal Membrane Oxygenation, Lactate Acid, Perfusion, Patient Discharge, Congenital Heart Defects

## Abstract

**Introduction:**

Findings of inadequate tissue perfusion might be used to predict the risk of mortality. In this study, we evaluated the effects of lactate and lactate clearance on mortality of patients who had undergone extracorporeal membrane oxygenation (ECMO).

**Methods:**

Patients younger than 18 years old and who needed venoarterial ECMO support after surgery for congenital heart defects, from July 2010 to January 2019, were retrospectively analyzed. Patients successfully weaned from ECMO constituted Group 1, and patients who could not be weaned from ECMO were in Group 2. Postoperative clinics and follow-ups of the groups including mortality and discharge rates were evaluated.

**Results:**

There were 1,844 congenital heart surgeries during the study period, and 55 patients that required ECMO support were included in the study. There was no statistically significant difference between the groups regarding demographics and operative variables. The sixth-, 12^th^-, and 24^th^-hour lactate levels in Group 1 were statistically significantly lower than those in Group 2 (P=0.046, P=0.024, and P<0.001, respectively). There were statistically significant differences regarding lactate clearance between the groups at the 24^th^ hour (P=0.009). The cutoff point for lactate level was found as ≥ 2.9, with 74.07% sensitivity and 78.57% specificity (P<0.001). The cutoff point for lactate clearance was determined as 69.44%, with 59.26% sensitivity and 78.57% specificity (P=0.003).

**Conclusion:**

Prognostic predictive factors are important to initiate advanced treatment modalities in patients with ECMO support. In this condition, lactate and lactate clearance might be used as a predictive marker.

## INTRODUCTION

Extracorporeal membrane oxygenation (ECMO) has been increasingly used in both adult and pediatric patients^[1]^. Approximately 2-3% of all pediatric patients undergoing cardiac surgery due to a congenital heart defect require ECMO support after surgery^[2]^. Despite the increasing experience and technological advances, high mortality and morbidity rates continue to be reported.

It is important to monitor whether adequate tissue perfusion is provided in order to predict mortality and morbidity in patients undergoing ECMO. The blood lactate level is a sign of anaerobic metabolism. It is a potential marker of inadequate tissue oxygenation. It was reported that high lactate levels, especially after cardiac surgery, are associated with increased morbidity and mortality^[3]^.

In this study, the effects of blood lactate level and lactate clearance on early-term mortality and morbidity of patients who underwent ECMO after pediatric cardiac surgery were evaluated.

## METHODS

Patients who underwent congenital heart surgery from July 2010 to January 2019 in our hospital were retrospectively analyzed. Among them, patients younger than 18 years old who required postoperative ECMO were included in the study. Patients older than 18 years old, patients who underwent veno-venous ECMO, cases requiring two or more ECMO procedures, and patients whose ECMO application ended in < 24 hours were excluded from the study. The present study protocol was approved by the review board of our institution (approval no.: ASM-EK-22/182). Demographic information, surgical and postoperative data, perfusion data, and cardiovascular intensive care unit (ICU) records were obtained from the patients’ files. The patients were divided into two groups - those who were successfully weaned from ECMO constituted Group 1, and those who could not be weaned from ECMO were in Group 2.

The indications for ECMO were low cardiac output syndrome (LCOS), inability to wean from cardiopulmonary bypass (CPB), and extracorporeal cardiopulmonary resuscitation (ECPR). Hypotension, end-organ failure (urine output < 0.5 mL/kg/h), metabolic acidosis, and cardiac index < 2 L/min/m^2^ despite maximum inotropic support were evaluated as LCOS.

Venoarterial ECMO was used in all patients. Direct intrathoracic cannulation was applied to all patients, those who underwent ECMO in the operating room and those who underwent ECMO in the ICU. The cannulation sites were the right or common atrium for venous outflow and the aorta or neo-aorta for arterial inflow. Cannulation techniques were modified according to the specific anatomical details, particularly in patients with preexisting cavo-pulmonary shunts. Systemic-to-pulmonary shunt was kept to preserve shunt patency during ECMO support. However, pulmonary blood flow was limited by adjusting the shunt diameter with a surgical clip in children with decreased systemic perfusion despite increased ECMO flow^[4]^. A standard ECMO circuit consisting of a cylinder pump head (Maquet Inc, Rastatt, Germany) and a membrane oxygenator (Medtronic Inc., Minneapolis, United States of America) was used.

ECMO circuits were prepared with blood in elective patients; circuits were prepared with crystalloid in ECPR patients. At the beginning of ECMO, 50 IU/kg heparin was administered, and then 10-25 IU/kg/hour heparin infusion was started to keep the activated coagulation time between 180 and 200 seconds. ECMO flow was adjusted based on patient’s variables such as systemic blood pressure, organ perfusion, and serial lactate measurements. Mechanical ventilation was maintained at minimum settings (respiratory rate: 10-12/min, positive end-expiratory pressure: 5-10 cmH₂O, fraction of inspired oxygen: 35-45%, and peak inspiratory pressure < 20 cmH₂O)^[5]^. All patients who received ECMO were sedated with benzodiazepine and an opioid. Inotropic support and ventilation support were kept at minimum levels during ECMO, and supports were gradually increased while weaning. The vasoactive inotropic score was calculated using the following formula: dopamine dose (mcg/kg/min) + dobutamine dose (mcg/kg/min) + 100 × epinephrine dose (mcg/kg/min) + 100 × norepinephrine dose (mcg/kg/min) + 10 × milrinone dose (mcg/kg/min) + 10.000 × vasopressin (U/kg/min)^[6]^.

Cardiac functions of the patients were evaluated by the same pediatric cardiologist via transthoracic echocardiography. In patients with adequate myocardial contraction and hemodynamic stability, weaning from ECMO was initiated with a flow rate < 25% of full flow. Successful ECMO weaning was assessed as the patient’s survival for > 24 hours after weaning from ECMO. In addition, ECMO was ended in cases of irreversible organ damage such as intracranial hemorrhage and lack of cardiovascular improvement.

### Statistical Analyses

IBM Corp. Released 2013, IBM SPSS Statistics for Windows, version 22.0, Armonk, NY: IBM Corp. program was used for statistical analysis. Compatibility of the parameters with the normal distribution was evaluated with the Kolmogorov-Smirnov test. In the comparison of quantitative data, Student’s *t*-test was used for the comparison of normally distributed parameters between two groups, and Mann-Whitney U test was used for comparisons between two groups of parameters that did not show normal distribution. Chi-square test, Fisher-Freeman-Halton test, and Yates’ correction for continuity were used to compare qualitative data. Receiver operating characteristic (ROC) curve was drawn to determine the cutoff point. Significance was evaluated at the *P*<0.05 level.

## RESULTS

There were 1,844 congenital heart surgeries during the study period, and ECMO was required in 3.5% (n=64) of them. After excluding three patients older than 18 years of age, four patients undergoing veno-venous ECMO, one patient who underwent ECMO more than twice, and one patient with an ECMO duration of < 24 hours, 55 patients were included in the study. There was no statistically significant difference between the groups in terms of demographic characteristics and preoperative and intraoperative data (Table 1).

**Table 1:** Patients’ demographics and perioperative variables.

Demographics and preoperative variables	Group 1 (n=27)	Group 2 (n=28)	*P*-value
Age (months)	27.8 ± 32	19.4 ± 41.5	0.4
Age group			
Newborn (0-30 days)	6 (22.2%)	11 (39.3%)	0.116
Infant (31-365 days)	7 (25.9%)	10 (35.7%)	
Child (˃ 365 days)	14 (51.9%)	7 (25%)	
Sex (male/female)	19-ago.	20-ago.	1.00
Weight (gram)	9437 ± 5757	7674 ± 8543	0.375
BSA (m^2^)	0.44 ± 0.21	0.35 ± 0.22	0.108
Ventricular physiology			
Biventricular repair (n)	25 (92.6%)	19 (67.9%)	0.055
Single ventricle (n)	2 (7.4%)	9 (32.1%)	
STAT category			
2	1 (3.7%)	2 (7.1%)	0.107
3	9 (33.3%)	7 (25%)	
4	15 (55.6%)	12 (42.9%)	
5	2 (7.4%)	7 (25%)	
*Before ECMO*			
Hemoglobin (mg/dl)	14.8 ± 2.9	14.6 ± 3.3	0.643
Hematocrit (%)	42.9 ± 8.2	43 ± 9	0.976
WBC (mcL)	12.5 ± 4.7	13.1 ± 4.4	0.659
Platelets (per mcl)	298 ± 128	248 ± 129	0.153
BUN (mg/dl)	12 ± 5.3	14.7 ± 5.4	0.069
Creatinine (mg/dl)	0.43 ± 0.2	0.54 ± 0.4	0.488
ALT (IU/l)	44.6 ± 68.1	46.9 ± 72	0.749
AST (IU/l)	60.2 ± 60.4	66.5 ± 64.3	0.372
CRP (mg/dl)	8.17 ± 8.61	5.82 ± 7.86	0.173
*During ECMO*			
Hemoglobin (mg/dl)	10.8 ± 0.9	11.3 ± 1.1	0.058
Hematocrit (%)	30.9 ± 2.6	32.1 ± 2.3	0.057
WBC (mcL)	13.3 ± 5.6	14.1 ± 7	0.633
Platelets (per mcL)	76 ± 33	65 ± 36	0.246
BUN (mg/dl)	27.3 ± 18.4	38.2 ± 18.1	0.03
Creatinine (mg/dl)	0.82 ± 0.7	1.1 ± 0.5	0.016
ALT (IU/l)	169 ± 310	190 ± 294	0.238
AST (IU/l)	446 ± 681	571 ± 894	0.148
CRP (mg/dl)	89.6 ± 65.9	78.7 ± 72.8	0.533
*Operative variables*			
Surgical correction			
Palliative (n)	6 (22.2%)	13 (46.4%)	0.109
Total correction (n)	21 (77.8%)	15 (53.6%)	
Cross-clamping time (min.)	83 ± 44.5	65.8 ± 40.7	0.063
CPB time (min.)	161.3 ± 87	126.9 ± 65	0.111
TCA (min.) (n=22)	25.73 ± 14.2	33.9 ± 16.4	0.224
*Postoperative variables*			
ECMO starting place			
Intensive care unit (n)	9 (33.3%)	17 (60.7%)	0.078
At operation (n)	18 (66.7%)	11 (39.3%)	
ECMO duration (days)	6 ± 5.1	8.9 ± 6.3	0.08
ECMO indication			
Unsuccessful weaning from CPB (n)	14 (51.9%)	11 (39.3%)	0.717
LCOS (n)	4 (14.8%)	5 (17.9%)	
ECPR (n)	9 (33.3%)	12 (42.9%)	
Vasoactive inotropic score	23.34 ± 15.6	19.1 ± 9.6	0.774
Reintubation	17 (63%)	8 (28.6%)	0.022
Peritoneal dialysis (n)	16 (59.3%)	23 (82.1%)	0.116
Peritoneal dialysis time (days)	10.8 ± 9.5	8.9 ± 5.8	0.575
Blood products (ml)	2279 ± 1461	2398 ± 1502	0.787
Postoperative exploration (n)	18 (66.7%)	14 (50%)	0.327
Mean lactate (mmol/L)	3.7 ± 2.3	6.2 ± 2.6	0.001
Intubation period (days)	24.7 ± 18	12.1 ± 7.2	0.004
ICU staying time (days)	30.4 ± 22.1	11.6 ± 7	0.000
Hospital staying time (days)	33.6 ± 22.1	11.8 ± 6.9	0.000

ALT=alanine aminotransferase; AST=aspartate aminotransferase; BSA=body surface area; BUN=blood urea nitrogen; CPB=cardiopulmonary bypass; CRP=C-reactive protein; ECMO=extracorporeal membrane oxygenation; ECPR=extracorporeal cardiopulmonary resuscitation; ICU=intensive care unit; LCOS=low cardiac output syndrome; STAT=STS-EACTS Congenital Heart Surgery Mortality Score; TCA=total circulatory arrest; WBC=white blood count

**Fig. 1:**
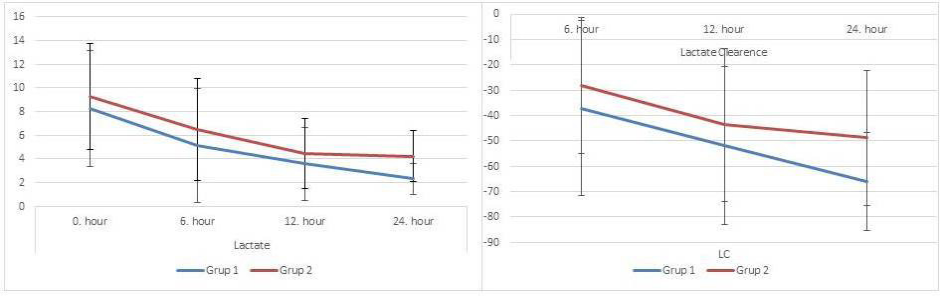
Differences of the lactate and lactate clearance levels in 24 hours.

Lactate, blood urea nitrogen, and creatinine levels in Group 1 were found to be statistically significantly lower than in Group 2 during ECMO (*P*=0.001, *P*=0.03, and *P*=0.016, respectively) (Table 1). In the evaluation of lactate levels, it was found that sixth-, 12^th^-, and 24^th^- hour lactate levels in Group 1 were statistically significantly lower than in Group 2 (*P*=0.046, *P*=0.024, and *P*<0.001, respectively). In terms of lactate clearance, there was no statistically significant difference between Group 1 and Group 2 in terms of the sixth hour and 12^th^ hour, and the difference between the groups reached statistically significance at 24 hours (*P*=0.009) (Table 2). In the comparisons of changes in lactate level and lactate clearance between time intervals within groups, statistically significant difference was found between 0 hour, six hours, 12 hours, and 24 hours in both groups (*P*=0.000) (Figure 1). Mean lactate levels (*P*=0.005), 24^th^-hour lactate levels (*P*=0.012), and 24^th^-hour lactate clearance (*P*=0.003) were found as independent risk factors for ECMO in Cox Regression model. While one-unit change in 24 hours of lactate increases the risk of ECMO by 0.632 times, one-unit change in mean lactate increases the risk of ECMO by 1.435 times.

**Table 2:** Evaluation of lactate levels between groups.

Lactate	Total (n=55)	Group 1 (n=27)	Group 2 (n=28)	*P*-value
0 hour	8.58 ± 4.35	8.28 ± 4.89	8.88 ± 3.83	0.469
6 hours	5.8 ± 4.54	5.13 ± 4.77	6.09 ± 3.04	0.046
12 hours	4.04 ± 3.03	3.59 ± 3.07	4.39 ± 2.67	0.024
24 hours	3.3 ± 2.02	2.32 ± 1.3	4.24 ± 2.17	< 0.001
Lactate clearance	Total (n=55)	Group 1 (n=27)	Group 2 (n=28)	*P*-value
6 hours	32.39 ± 31.20	37.13 ± 34.42	27.84 ± 27.65	0.148
12 hours	46.7 ± 32.61	51.81 ± 31.21	41.70 ± 33.78	0.148
24 hours	56.56 ± 25.52	66.11 ± 19.18	47.35 ± 27.73	0.007

**Fig. 2:**
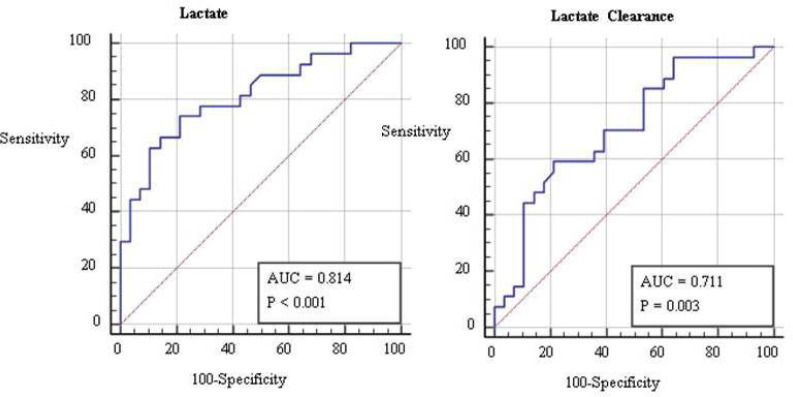
Receiver operating characteristic curve to demonstrate the predictive value of lactate and lactate clearance levels at the 24^th^ hour. AUC=area under the curve.

The area under the curve was calculated by ROC analysis. The cutoff point determined for the lactate level was ≥ 2.9. The sensitivity of this value was found to be 74.07%, and the specificity was 78.57% (95% confidence interval [CI] 0.686-0.906, *P*<0.001). The cutoff point determined for the lactate clearance was 69.44%. The sensitivity of this value was found to be 59.26%, and the specificity was 78.57% (95% CI 0.573-0.825, *P*=0.003) (Figure 2).

Pulmonary complications, such as longer ventilation that needed to tracheostomy, chylothorax, and pulmonary hypertension, occurred in 11% of the patients. Bleeding was observed in 11 (20%) patients. The rate of renal complications was 22%, and peritoneal dialysis was performed in 39 patients (Table 3). Although successful weaning from ECMO, 12 patients died in Group 1 during the hospitalization period. The causes of the mortality were neurological complications in three patients, bleeding in one patient, hepatorenal syndrome in one patient, cardiac problems in two patients, and sepsis in five patients. The discharge rate of Group 1 was 56%.

**Table 3:** Complications of extracorporeal membrane oxygenation according to the Extracorporeal Life Support Organization.

Complications	All (n=55)	Group 1 (n=27)	Group 2 (n=28)
Mechanical (n)	8 (14.5%)	2 (7.4%)	6 (21.4%)
Pulmonary (n)	6 (11%)	4 (14.8%)	2 (7.1%)
Neurological (n)	4 (7.3%)	4 (14.8%)	0
Hemorrhagic (n)	11 (20%)	6 (22.2%)	5 (17.9%)
Renal (n)	12 (21.8%)	5 (18.5%)	7 (25%)
Infection (n)	24 (43.6%)	16 (59.3%)	8 (28.6%)
Gastrointestinal (n)	6 (11%)	2 (7.4%)	4 (14.3%)

## DISCUSSION

In this study, we revealed that postoperative early-term lactate and lactate clearance levels might be predictive factors for the risk of mortality in patients with ECMO. The cutoff point was 2.9 mmol/L. As a result, we could determine lactate levels > 2.9 mmol/L as a predictive factor for mortality.

Renal failure, stroke, disseminated intravascular coagulopathy, single ventricle, longer CPB times, elevated lactate levels in the first 72 hours, and higher inotrope score (> 2000) are reported as risk factors for mortality during ECMO^[7,8]^. Additionally, inability to obtain negative fluid balance, high serum lactate levels, and high total bilirubin in the first day of ECMO was found to be an independent risk factor for the weaning^[4]^. Yang et al.^[9]^ declared that hyperlactatemia and prolonged prothrombin time ( > 6 seconds) were associated with increased mortality in 30 days. In our retrospective study, we revealed that lactate and lactate clearance might be used as predictive factors during ECMO.

Low weight has negative effects on survival in pediatric cardiac surgery^[5,10]^. It was reported that patients younger than 10.6 months of age and weighing < 5.5 kg are associated with higher mortality^[4]^. In addition to age and sex, early lactate behavior during ECMO is highly associated with in-hospital mortality, and early lactate levels might predict the success of ECMO^[11]^. Half of our patients in Group 1 were older than one years old, however there was no statistically significant difference between the groups in terms of age and weight.

Lactate is a marker that is influenced by macro and microcirculation^[11]^. Higher lactate clearance improves tissue perfusion, decreasing lactate levels^[12]^. Lactate clearance was defined as a prognostic factor in pediatric patients with ECMO in the first 24 hours^[12]^. After initiation of ECMO, lactate and lactate clearance levels are predictive factors for mortality since these are the signs of tissue perfusion and organ damage in 24 to 48 hours^[3,10,11,13]^. Mean lactate levels and decreased lactate clearance in the first six and 12 hours were reported as worse prognostic factors^[11]^. While lactate levels before ECMO have no effect on mortality, lactate levels ˃ 5 mmol/L during ECMO are associated with increased mortality^[5]^. In other aspect, Baslaim et al.^[8]^ declared that lactate level ˃ 9 mmol/L as a predictive value for mortality. Cutoff points of lactate clearance in three, nine, and 12 hours were reported as 3.8%, 51%, and 56%, respectively^[12]^. In our ROC analyses, cutoff points were 2.9 mmol/L for lactate and 69.44% for lactate clearance.

Increased lactate levels might be the sign of residual lesions after surgery^[3]^. In the presence of residual cardiac defects, weaning from ECMO might not be managed^[14]^. Early treatment of the residual lesions, especially in three days, improves the outcomes^[7,15]^. Kuraim et al.^[7]^ reported that 40% of the patients who necessitated ECMO have residual defect, and they were treated by endovascular or surgical approach. In our study, there was no difference between the groups regarding reoperation.

Bleeding necessitating reoperation and transfusion of blood products is another risk factor for mortality^[10,16]^. Blood products and sepsis increase the risk of mortality after weaning from ECMO^[13]^. In our study, there was no difference between the groups regarding need for blood products.

Dohain et al.^[17]^ reported a 40% survival rate with ECMO, and they declared that the survival rate was higher with biventricular repair. Single ventricular repair is declared as a risk factor for mortality^[5,18,19]^. Although in our study the survival rate with biventricular repair and single ventricular repair were 56.8% and 18.2%, respectively, it did not reach statistically significance (*P*=0.055). This might be caused by the number of patients in the groups and might reach statistically significance in further studies.

In some studies, it was reported that ECMO initiation in the operating room has higher survival rates^[17]^. Starting the ECMO after resuscitation might increase the mortality risk to 80%^[19]^. In contrast, it was reported that the indication of ECMO has no effect on mortality^[13,14]^. The interval between the operation and ECMO might increase ECMO duration and hospital and ICU length of stay, however the mortality rate is similar in patients who weaned from ECMO at the operating room^[20]^. Similarly, we could not find any difference on survival regarding indication of ECMO.

Longer duration of ECMO was reported as a risk factor for mortality^[4]^. In different studies, seven days or nine days were reported as cutoff points for increased mortality^[4,5]^. Every extra day beyond seven days has increased the mortality risk by 12%^[17]^. In our series, 44% of the patients were on ECMO for more than seven days. The survival of patients with < 7 days of ECMO was 65%, and survival of patients with ≥ 7 days of ECMO was 29%.

On ECMO, acute renal failure is a risk factor for mortality^[10,13]^. However, in this clinical condition, it is difficult to say which develops first^[17]^. Renal failure might develop due to hypoperfusion or bleeding^[15]^. Effective diuresis helps to decrease the myocardial stress^[16]^. Early fluid removal with peritoneal dialysis or hemofiltration helps to improve renal function^[10,19]^. In our patients, kidney function was found to be a risk factor for mortality.

During ECMO, the risk of mechanical, hemorrhagic, neurological, pulmonary, renal, cardiac, and infectious complications were reported as 9-46%, 37-60%, 21-25%, 8-20%, 43-53%, 73-99%, and 8-39%, respectively^[5]^. Survival rate of ECMO after the pediatric cardiac surgery was found to be 37%^[14]^. In a review of a 10-year period, it was reported that after weaning from ECMO, discharge rate was 65%, and 51% of these patients were alive in the five-year follow-up period^[7]^. In a study, Ergun et al.^[5]^ reported that they weaned 60% of their patients from ECMO, however, they discharged 38% of the patients. In our study, the complication rate is comparable with the literature and was similar between the groups. We discharged more than half of our patients (55.6%) after the weaning.

### Limitations

This study has some limitations, including deficiencies related to retrospective studies. We evaluated all patients younger than 18 years old and who needed ECMO support. We studied the effects of lactate levels for postoperative mortality in the patients with ECMO. It is known that lonely ECMO is a predictive factor for mortality after cardiac surgery. The patient population was heterogeneous, including infants to 17-year-old patients. However, all patients were operated due to congenital heart defects, and operative procedures were similar between the groups. Further prospective studies in different age groups and larger patient groups might be helpful to evaluate the effects of lactate and lactate clearance during ECMO.

## CONCLUSION

ECMO support, which is a life-saving procedure during congenital cardiac surgery, has been associated with high morbidity and mortality rates. Monitoring blood lactate level and lactate clearance during ECMO is valuable not only as a marker of morbidity but also to predict mortality. Levels of lactate and lactate clearance at the 24^th^ hour during ECMO might be used as prognostic prediction markers.

**Table:** 

Authors’ Roles & Responsibilities	
TA	Substantial contributions to the conception or design of the work; and the analysis of data for the work; drafting the work and revising it critically for important intellectual content; final approval of the version to be published
AHA	Substantial contributions to the conception or design of the work; and the analysis of data for the work; revising the work critically for important intellectual content; final approval of the version to be published
MU	Substantial contributions to the analysis and interpretation of data for the work; drafting the work; final approval of the version to be published
HU	Substantial contributions to the analysis of data for the work; revising the work critically for important intellectual content; final approval of the version to be published
